# Solution for Determining Modulus of Elasticity of Natural Materials Using Vibrations of Non-Uniform Circular Cross-Section Cantilevers

**DOI:** 10.3390/ma16103868

**Published:** 2023-05-21

**Authors:** Jerzy Podgórski, Bartosz Kawecki

**Affiliations:** Faculty of Civil Engineering and Architecture, Lublin University of Technology, ul. Nadbystrzycka 40, 20-618 Lublin, Poland; j.podgorski@pollub.pl

**Keywords:** natural materials properties, plant stems experimental testing, non-uniform bar vibrations, solution by Bessel functions, tapered pipe vibrations, eigenfrequency calculation, modulus of elasticity determination

## Abstract

The article presents an original method for determining the modulus of elasticity of natural materials. A studied solution was based on vibrations of non-uniform circular cross-section cantilevers solved using Bessel functions. The derived equations, together with experimental tests, allowed for calculating the material’s properties. Assessments were based on the measurement of the free-end oscillations in time using the Digital Image Correlation (DIC) method. They were induced manually and positioned at the end of a cantilever and monitored in time using a fast Vision Research Phantom v12.1 Camera with 1000 fps. GOM Correlate software tools were then used to find increments of deflection on a free end in every frame. It provided us with the ability to make diagrams containing a displacement–time relation. To find natural vibration frequencies, fast Fourier transform (FFT) analyses were conducted. The correctness of the proposed method was compared with a three-point bending test performed on a Zwick/Roell Z2.5 testing machine. The presented solution generates trustworthy results and can provide a method to confirm the elastic properties of natural materials obtained in various experimental tests.

## 1. Introduction

The properties of a material can be identified using various experimental methods [1,2,3]. One primary property, the modulus of elasticity, can be evaluated by obtaining natural vibration frequencies in simple static schemes. When using clamped–free boundary conditions, the first natural frequency can be determined by measuring oscillations at the cantilever’s free end. The Digital Image Correlation (DIC) technique [4] is valuable, because vibration can be assessed without deploying other sensors, which can result in increased concentrated mass. With moderately light samples, this approach is essential. Plant stems or other natural materials [5,6] belong to this group. The subsequent case we present is one in which the cross section may differ along the length of the tested element. Simplifications are often introduced by averaging dimensions, which may generate calculation errors for non-uniform sections. In consequence, this may lead to under- or overestimation of mechanical properties. Thus, diverse sections should be incorporated when evaluating characteristics of the elements in experimental tests. The authors of the paper consider a cross section’s non-uniformity in calculations and examine its influence compared to the results achieved with simplified cross-section approximations.

Because of the native characteristics of the elements examined, the cross-sectional divergences, which led to the development of model plants, are especially significant. Such structures often have circular-symmetric cross-sections with tapering along the length. Solid circular, or pipe, cross sections are the most common choice for modelling slender bars with variable stiffness, and capture the behaviour of plant elements reliably well. The challenge of determining the eigenfrequencies of such bars, despite the long history of such endeavours, still fascinates researchers, who advance solutions to this task using multiple methods. These are analytical (exact or approximate) or, in more recent times, numerical, wherein the most typical choice is the finite element method or, much less often, the finite difference method.

Conceivably, the first solution to the problem of vibration of a rod with a circular cross section of linearly varying diameter was proposed by Kirchhoff [7] for a clamped–free scheme. He calculated the first natural frequency and the amplitude of oscillation using the power series method to solve the differential equation and compared these results to the quantities obtained for a rod with a constant circular cross section. The accuracy of this solution (four correct significant digits) was exact, considering the computational capabilities at his disposal in the late 19th century. Subsequent papers describing a similar problem appeared in the early 20th century. Nicholson [8] and Wrinch [9] implemented Bessel functions in their solutions. Wrinch, similarly to Kirchhoff, studied the vibration of a cone fixed at the base and calculated several eigenfrequencies utilising the results of Airey [10], who obtained them by examining the vibrations of circular plates.

Similar problems were often solved by the Ritz–Galerkin method in the second half of the 20th century. Significant achievements in its applications were made by Rao [11], who solved the equations of motion of cantilever bars with a rectangular tapered cross-section. Computational methods were applied too. Numerical integration of the equation of transverse vibration of a Bernoulli–Euler beam was performed by Mabie and Rogers [12], who determined the free vibration frequencies of a beam with a linearly double-tapered rectangular section. The equation of motion was numerically integrated; however, details about the method were not provided. The boundary conditions described in the paper correspond to a clamped–simply supported beam, but the authors mention the cantilever scheme in the introduction.

Abdelghany et al. [13] analysed three examples of circular section members implementing several boundary conditions, such as simply supported, clamped–clamped and clamped–roller beam. They employed the differential transformation method. A 3D analysis was performed by Kang and Leissa [14], who examined thick, tapered circular section bars. Results were shown for nine different cases with linear, quadratic and cubic variations of radial thickness. Jaworski [15] utilised the Rayleigh approach to investigate a cantilever column as a truncated cone and a hollow truncated cone. An analytical solution was reached for the first form of the vibrations’ eigenvalue. A differential quadrature method was used, among others, by, Al Kaisy et al. [16], who studied a general non-uniform bar, whereas De Rosa et al. [17] attempted to derive the frequency–axial load relationship for a variable circular section beam. In a preceding paper [18], these authors analysed the dynamic characteristics of beams with a linearly changing cross section with elastic support at the ends. The equation of motion was solved using Bessel functions. The article includes full expressions for the boundary conditions that led to the eigenfrequencies.

Bessel functions were used, among others, by Li [19], who studied non-uniform shear beams with an arbitrary distribution of mass or stiffness. Taha and Abohadima [20] considered non-uniform viscoelastic flexural structures and attained results for a wide range of their characteristics. Kisa and Gurel [21] analysed circular section members containing non-propagating open cracks. Three numerical examples were given to investigate the effects of location and depth of cracks. Attarnejad and Shahba [22] studied revolved non-prismatic cantilevers. 

Lee Jung and Lee Youn [23] used the transfer matrix and Frobenius methods to find the values and eigenvectors of a bar with a linearly varying section height. They noticed that the number of terms of the series which must be summed up to obtain the required accuracy strongly depends on the cross-section taper ratio. The results were subsequently discussed by Banerjee and Ananthapuvirajah [24], who applied Bessel functions in their solution, proving the greater efficiency of the method.

Li et al. [25] analysed the free longitudinal vibration of a circular truncated nanocone based on the theory of non-local elasticity. The results apply to the design of sensors and oscillators based on circular truncated nanocones. Nguyen Thi Nhung et al. [26] used the perturbation method and finite element analysis for the stochastic natural frequency problem of inhomogeneous beams with a random elastic modulus field. Their findings showed that the mesh density did not affect the natural frequency significantly.

Due to a vast number of related studies, the preceding review was limited to a few works. Furthermore, some of them contain an extensive bibliography, enabling the expansion of the topic under consideration.

## 2. Materials and Methods

### 2.1. Motivation for the Approach

**Figure 1:**
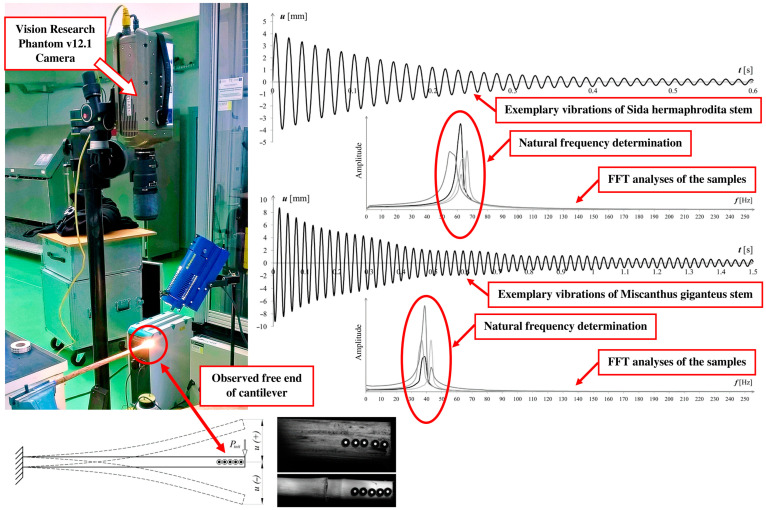
Natural vibration frequency testing method proposed by the authors.

The solution for a cantilever beam with a non-uniform cross section can be used for many purposes. One of them can be a reverse method of exact determination of mechanical properties in natural plant stems or other materials undergoing non-uniformity of the cross section. An example is the method suggested, but not explained thoroughly, by the authors in our earlier works [1,2]. The determination of the modulus of elasticity, being a main goal of a procedure, was based on the first plant stems’ cantilevers’ eigenfrequency. Vibrations were induced manually and oscillations of the free end of the cantilever were measured in time using a fast Vision Research Phantom v12.1 Camera with 1000 fps. GOM Correlate software was then used to read increments of deflection *u*(+) and *u*(−) on a free end in every frame. It enabled us to create diagrams containing a displacement–time relation. In order to find the first eigenfrequency, fast Fourier transform (FFT) analyses were performed (Figure 1).

The free vibration frequency of a prismatic bar can be determined by the well-known [27,28] Formula (1):

(1) $$\omega =\frac{\beta^{2}}{l^{2}}\sqrt{\frac{Eℑ}{\varrho A}}$$

where:

ω —the angular frequency [rad/s];
ϱ —mass density;
A —area of the cross section;
ℑ —moment of inertia of the cross section;
E —elastic modulus (Young’s modulus);
l —length of the bar;
β —the coefficient depends on the boundary conditions of the bar.

After small rearrangements, Formula (1) can be written as (2):

(2) $$\omega =2\pi f\;\to \;E={\left(\frac{2\pi f}{\beta^{2}}\right)}^{2}\frac{\varrho Al^{4}}{ℑ}$$

where f is a natural frequency [Hz].

Boundary conditions of an analysed bar lead to the so-called transcendental equation, which has an infinite number of roots. The highest accuracy can be obtained for the first (lowest) free vibration frequency. For a prismatic cantilever bar, an equation can be used with the form $cosh\left(\beta \right)cos\left(\beta \right)+1=0$ [27,28], and the first root of this equation has the value β≅1.8751 . Calculating the Young’s modulus is then possible using Equation (2), after determining the vibration frequency of a tested material.

Analysing a bar with variable stiffness, the problem is to define a correct value of the β -factor. In many investigations, this issue is often simplified by accepting the β -factor as for a constant-stiffness bar. In the authors’ opinion, this may lead to significant calculation errors. Thus, the subsequent parts of the paper focus on incorporating non-uniformity of the sample section and discussing difficulties that occur during calculations.

### 2.2. Theoretical Introduction to the Approach

**Figure 2:**
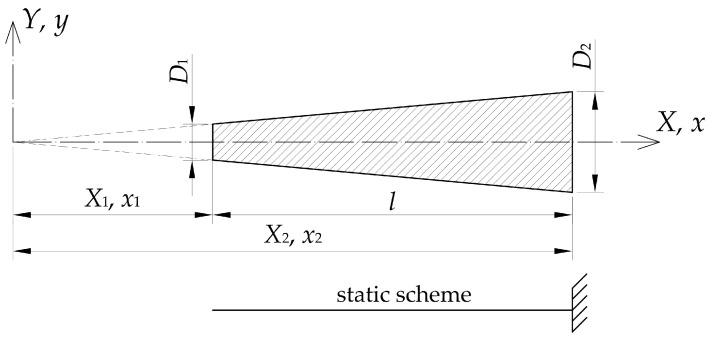
Denotations used in the theoretical approach assumptions.

The equation describing the free undamped flexural vibration of the Euler–Bernoulli bar, shown in Figure 2, can be written as follows (3) [28]:

(3) $$\frac{d^{2}}{dX^{2}}\left(Eℑ\left(X\right)\frac{d^{2}Y}{dX^{2}}\right)+\mu \left(X\right)\frac{d^{2}Y}{dt^{2}}=0$$

where E denotes the modulus of elasticity (Young’s modulus), ℑ is the moment of inertia of the cross section, and μ is the mass per unit length of the bar. After taking a dimensionless coordinate, x=X/l , and assuming a solution in the form (4):

(4) $$Y\left(x,t\right)=ly\left(x\right)\sin\left(\omega t\right)$$

an equation describing the transverse displacement of the bar is obtained (5):

(5) $$\left[\;\frac{1}{l^{4}}\frac{d^{2}}{dx^{2}}\left(Eℑ\left(x\right)\frac{d^{2}y\left(x\right)}{dx^{2}}\right)-\mu \left(x\right)\omega^{2}y\left(x\right)\right]\sin\left(\omega t\right)=0$$

Since the natural frequency is of interest, the problem can be reduced to solving an ordinary differential Equation (6):

(6) $$\frac{d^{2}}{dx^{2}}\left(ℑ\left(x\right)\frac{d^{2}y\left(x\right)}{dx^{2}}\right)-\frac{\varrho A\left(x\right)\omega^{2}l^{4}}{E}y\left(x\right)=0$$

where $\mu \left(x\right)=\varrho A\left(x\right)$ and $A\left(x\right)$ is the cross-sectional area and ϱ is the material density.

Characteristics of a bar cross section, $ℑ\left(x\right)$ and $A\left(x\right)$ , for many types of variation in bar geometry, can be presented in the form (7)—(cf. Appendix A):

(7) $$ℑ\left(x\right)={ℑ}_{o}x^{n+2},\;\;\;A\left(x\right)=A_{o}x^{n}$$

where:

n=1 —for a tapered pipe of a constant thickness or wedge with a fixed width;
n=2 —for a truncated cone or a tapered pipe of a variable thickness, as well as a pyramid with rectangular cross section.

An explanation of these values is provided in the next section and in Appendix A.

After taking into account the relation (7), Formula (8) is obtained:

(8) $$\frac{d^{2}}{dx^{2}}\left(x^{n+2}\frac{d^{2}y}{dx^{2}}\right)-\eta^{4}x^{n}y=0$$

where: $\eta^{4}=\frac{\rho A_{o}\omega^{2}l^{4}}{E{ℑ}_{o}}$ .

After minor transformations, Equation (8) can be presented in a compact form (9), as in [29]:

(9) $$\left(x\frac{d^{2}}{dx^{2}}+\left(1+n\right)\frac{d}{dx}+\eta^{2}\right)\left(x\frac{d^{2}}{dx^{2}}+\left(1+n\right)\frac{d}{dx}-\eta^{2}\right)y=0$$

The solution of this equation is the sum $\left(y=y_{1}+y_{2}\right)$ of the solutions of two simpler relationships (10):

(10) $$x\frac{d^{2}y_{1}}{dx^{2}}+\left(1+n\right)\frac{dy_{1}}{dx}+\eta^{2}y_{1}=0\;\;\;\mathrm{and}\;\;\;x\frac{d^{2}y_{2}}{dx^{2}}+\left(1+n\right)\frac{dy_{2}}{dx}-\eta^{2}y_{2}=0$$

The functions $y_{1}\left(x\right)$ and $y_{2}\left(x\right)$ , which are solutions of these equations, can be represented as linear combinations of Bessel functions (11) and (12):

(11) $$y_{1}=\frac{1}{\sqrt{x^{n}}}\left[C_{1}J_{n}\left(2\eta \sqrt{x}\right)+C_{2}Y_{n}\left(2\eta \sqrt{x}\right)\right]$$

(12) $$y_{2}=\frac{1}{\sqrt{x^{n}}}\left[C_{3}I_{n}\left(2\eta \sqrt{x}\right)+C_{4}K_{n}\left(2\eta \sqrt{x}\right)\right]$$

where:

$J_{n}$ —Bessel function of the first kind of order n ;
$Y_{n}$ —Bessel function of the second kind of order n ;
$I_{n}$ —modified Bessel function of the first kind of order n ;
$K_{n}$ —modified Bessel function of the second kind of order n ;
$C_{1},\;C_{2},\;C_{3}$ and $C_{4}$ are constants.

For the convenience of further transformations, a new variable $\xi =2\eta \sqrt{x}$ is introduced, which leads to (13) and (14):

(13) $$\xi^{2}=4\eta^{2}x,\;\;\;x=\frac{1}{4\eta^{2}}\xi^{2},\;\;\;\;\;dx=\frac{1}{2\eta^{2}}\xi d\xi ,\;\;x^{n}={\left(\frac{1}{2\eta }\xi \right)}^{n+2}$$

(14) $$y={\left(\frac{2\eta }{\xi }\right)}^{n}\left[C_{1}J_{n}\left(\xi \right)+C_{2}Y_{n}\left(\xi \right)+C_{3}I_{n}\left(\xi \right)+C_{4}K_{n}\left(\xi \right)\right],\;\;\;\frac{dy}{dx}=\frac{2\eta^{2}}{\xi }\frac{dy}{d\xi }$$

$C_{1},\;C_{2},\;C_{3}$ and $C_{4}$ are constants that should be chosen in order to satisfy the boundary conditions of the problem. At the fully restrained end, they are (15):

(15) $$X=X_{2}\;\to \;\;\frac{dY}{dX}=0,\;\;\;Y=0\;$$

and at the free end (16):

(16) $$X=X_{1}\;\to \;\;\frac{d^{2}Y}{dX^{2}}=0,\;\;\;\frac{d^{3}Y}{dX^{3}}=0$$

which leads to the equivalent conditions (17a,b):

(17a) $$x=x_{2}\;\to \;\;y=0,\;\;\;\frac{dy}{dx}=0$$

(17b) $$x=x_{1}\;\to \;\;\frac{d^{2}y}{dx^{2}}=0,\;\;\;\frac{d^{3}y}{dx^{3}}=0$$

After applying the recursive formulas for Bessel functions [30] and small transformations, Equations (18) and (19) are obtained:

(18) $$y={\left(\frac{2\eta }{\xi \;}\right)}^{n}\left[C_{1}J_{n}\left(\xi \right)+C_{2}Y_{n}\left(\xi \right)+C_{3}I_{n}\left(\xi \right)+C_{4}K_{n}\left(\xi \right)\right]$$

(19) $$\frac{dy}{dx}=-\eta {\left(\frac{2\eta }{\xi \;}\right)}^{n+1}\left(C_{1}J_{n+1}+C_{2}Y_{n+1}-C_{3}I_{n+1}+C_{4}K_{n+1}\right)$$

Boundary conditions (17a) are equivalent to the conditions (20a,b):

(20a) $$C_{1}J_{n}\left(\xi_{2}\right)+C_{2}Y_{n}\left(\xi_{2}\right)+C_{3}I_{n}\left(\xi_{2}\right)+C_{4}K_{n}\left(\xi_{2}\right)=0$$

(20b) $$C_{1}J_{n+1}\left(\xi_{2}\right)+C_{2}Y_{n+1}\left(\xi_{2}\right)-C_{3}I_{n+1}\left(\xi_{2}\right)+C_{4}K_{n+1}\left(\xi_{2}\right)=0$$

Conditions (17b) lead to the Equations (21a,b):

(21a) $$C_{1}J_{n+2}\left(\xi_{1}\right)+C_{2}Y_{n+2}\left(\xi_{1}\right)+C_{3}I_{n+2}\left(\xi_{1}\right)+C_{4}K_{n+2}\left(\xi_{1}\right)=0\;$$

(21b) $$C_{1}J_{n+3}\left(\xi_{1}\right)+C_{2}Y_{n+3}\left(\xi_{1}\right)-C_{3}I_{n+3}\left(\xi_{1}\right)+C_{4}K_{n+3}\left(\xi_{1}\right)=0$$

After introducing substitutions (22):

(22) $$\xi_{1}=2\eta \sqrt{x_{1}}=\eta \zeta_{1}\to \zeta_{1}=2\sqrt{x_{1}},\;\;\xi_{2}=2\eta \sqrt{x_{2}}=\eta \zeta_{2}\to \zeta_{2}=2\sqrt{x_{2}}$$

the system of Equations (20) and (21) may be written in a matrix form as (23):

(23) $$\left[\begin{array}{cccc} J_{n}\left(\eta \zeta_{2}\right) & Y_{n}\left(\eta \zeta_{2}\right) & I_{n}\left(\eta \zeta_{2}\right) & K_{n}\left(\eta \zeta_{2}\right)\\ J_{n+1}\left(\eta \zeta_{2}\right) & Y_{n+1}\left(\eta \zeta_{2}\right) & -I_{n+1}\left(\eta \zeta_{2}\right) & K_{n+1}\left(\eta \zeta_{2}\right)\\ J_{n+2}\left(\eta \zeta_{1}\right) & Y_{n+2}\left(\eta \zeta_{1}\right) & I_{n+2}\left(\eta \zeta_{1}\right) & K_{n+2}\left(\eta \zeta_{1}\right)\\ J_{n+3}\left(\eta \zeta_{1}\right) & Y_{n+3}\left(\eta \zeta_{1}\right) & -I_{n+3}\left(\eta \zeta_{1}\right) & K_{n+3}\left(\eta \zeta_{1}\right) \end{array}\right]\cdot \left[\begin{array}{c} C_{1}\\ C_{2}\\ C_{3}\\ C_{4} \end{array}\right]=\left[\begin{array}{c} 0\\ 0\\ 0\\ 0 \end{array}\right]\to B\left(\eta \right)\cdot c=0$$

The above leads to the condition of zeroing the determinant (24):

(24) $$\left|\begin{array}{c} B\left(\eta \right) \end{array}\right|=0$$

Eigenvalues $\eta_{i}$ determined using the condition (24) enable us to calculate natural vibration frequencies (25):

(25) $$\eta_{i}^{4}=\frac{\varrho A_{o}\omega_{i}^{2}l^{4}}{E{ℑ}_{o}}\;\to \;\omega_{i}=\frac{\eta_{i}^{2}}{l^{2}}\sqrt{\frac{E{ℑ}_{o}}{\varrho A_{o}}}$$

Inserting $A_{2}$ and ${ℑ}_{2}$ instead of $A_{o}$ and ${ℑ}_{o}$ (the bar section parameters at the clamping point)—(cf. Appendix A), where $x=x_{2}$ , Formula (26) is obtained:

(26) $$\eta_{i}^{4}=\frac{\varrho A_{2}x_{2}{}^{2}\omega_{i}^{2}l^{4}}{E{ℑ}_{2}}\;\to \;\omega_{i}=\frac{\eta_{i}^{2}}{\;x_{2}l^{2}}\sqrt{\frac{E{ℑ}_{2}}{\varrho A_{2}}}$$

Comparing Formula (26) with Equation (1), a relationship between the eigenvalue η and the searched-for β -factor is obtained, which results in the vibration frequency of the non-uniform bar: $\beta^{2}=\eta^{2}/x_{2}$ .

## 3. Results and Discussion

The main goal of the examples presented was to study an effect of cross-sectional variations on the first natural frequency of a bar fixed at the “thicker” end. This allowed us to find the correct value of the β -factor (cf. Equations (1) and (2)), necessary for estimating the Young’s modulus of the material under consideration. Two examples were included in the calculations: (1) a tapered pipe of a constant wall thickness, (2) a truncated cone and a tapered pipe of linearly varying thickness. Assumptions which should be introduced into the model are given subsequently.

In all presented calculations, the examples assumed:

- Bar length: l=500 mm ;
- External diameter of the bar at the support: $D_{2}=14\;\mathrm{mm}$ ;
- Thickness of the pipe wall at the support, in the case of linearly varying thickness: $g_{2}=3\;\mathrm{mm}$ ;
- Constant thickness of the tapered pipe: g=2 mm .

A tapering coefficient of a cross section was defined as: $\vartheta =1-D_{1}/D_{2}$ , which varies from 0, for a prismatic bar to 1 for a conical bar.

### 3.1. Tapered Pipe with Constant Wall Thickness

Taking the value of the tapering coefficient ϑ , Formula (27) is obtained:

(27) $$x_{2}=\vartheta^{-1},\;\;x_{1}=x_{2}-1,\;\;\zeta_{1}=2\sqrt{x_{1}},\;\;\zeta_{2}=2\sqrt{x_{2\;}}$$

With the cross-sectional variation considered, the exponent is n=1 (cf. Appendix A.1), so the boundary conditions matrix (Equation (23)) takes the form (28):

(28) $$B\left(\eta \right)=\left[\begin{array}{cccc} J_{1}\left(\eta \zeta_{2}\right) & Y_{1}\left(\eta \zeta_{2}\right) & I_{1}\left(\eta \zeta_{2}\right) & K_{1}\left(\eta \zeta_{2}\right)\\ J_{2}\left(\eta \zeta_{2}\right) & Y_{2}\left(\eta \zeta_{2}\right) & -I_{2}\left(\eta \zeta_{2}\right) & K_{2}\left(\eta \zeta_{2}\right)\\ J_{3}\left(\eta \zeta_{1}\right) & Y_{3}\left(\eta \zeta_{1}\right) & I_{3}\left(\eta \zeta_{1}\right) & K_{3}\left(\eta \zeta_{1}\right)\\ J_{4}\left(\eta \zeta_{1}\right) & Y_{4}\left(\eta \zeta_{1}\right) & -I_{4}\left(\eta \zeta_{1}\right) & K_{4}\left(\eta \zeta_{1}\right) \end{array}\right]$$

Equating a determinant of the matrix B to zero enables us to find its eigenvalues $\eta_{i}$ :

(29) $$\det\left(B\left(\eta \right)\right)=0\;\to \eta_{1},\;\eta_{2},\;\eta_{3}\ldots \;\eta_{\infty }\;\to \;\beta =\eta_{1}\sqrt{\vartheta }$$

Since determining an elastic modulus (Equation (2)) is of interest, it is enough to calculate only the first eigenvalue $\eta_{1}$ , which will give the requested parameter $\beta =\eta_{1}/\sqrt{x_{2}}$ . The method described gives $x_{2},x_{1}\to \infty$ for a bar with constant thickness when ϑ=0 . Because of that finding, this value requires using a different approach. Thus, a classical equation is used [27,28]: $\cosh\left(\beta \right)\cos\left(\beta \right)+1=0$ , which gives the first eigenvalue β≅1.8751 . For a full (conical) taper of the pipe, ϑ=1 , so $x_{2}=1$ , $x_{1}=0$ , which also gives $\zeta_{1}=0,\;\zeta_{2}=2$ . Such a value of $\zeta_{1}$ results in infinite values of the Bessel functions $Y\left(\zeta_{1}\right)$ and $K\left(\zeta_{1}\right)$ , which results in unlimited displacements of the bar end. Therefore, zero values of constants $C_{2}$ and $C_{4}$ in Equations (20) and (21) should be assumed and only Equations (20a) and (20b) should be solved. Boundary conditions for the free end of a bar (Equation (21)) are satisfied automatically, because the functions $J_{3}\left(\zeta \right),\;J_{4}\left(\zeta \right)$ and $I_{3}\left(\zeta \right),\;I_{4}\left(\zeta \right)$ describing them have zero values at $\zeta_{1}=0$ . Subsequently, a simple equation is obtained: $J_{1}\left(2\eta \right)I_{2}\left(2\eta \right)+J_{2}\left(2\eta \right)I_{1}\left(2\eta \right)=0$ , whose solution is η≅2.3054 .

Table 1 shows the results of the eigenvalue search, calculated for many values of the tapering coefficient ϑ in the range 0≤ϑ≤1 , and $D_{2}=14\;\mathrm{mm}$ , g=2 mm , l=500 mm . The values marked in red do not apply to the pipe, since at this tapering and wall thickness, the end is filled completely. The values highlighted in blue indicate a significant error ( ε ) in the approximation of the moment of inertia at the end of the bar. The ${ℑ}_{1}$ here has a value more than 10% less than the exact value. An explanation of the cause and magnitude of this error can be found in Appendix A.1 (Equation (A2)). All values in the table are obviously correct for a constant-width wedge.

**Figure 3:**
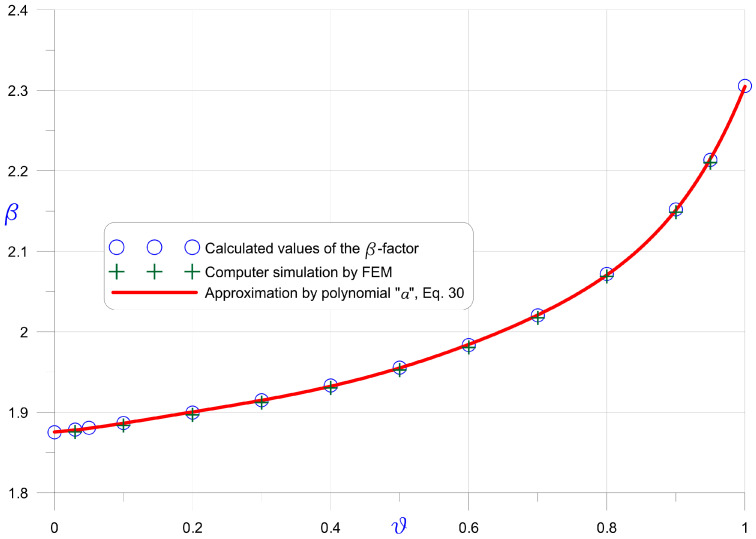
β -factor in dependence on the pipe tapering coefficient ϑ for tapered pipe with constant wall thickness (compare Figure A1) and constant-width wedge (compare Figure A2).

Figure 3 shows the results simultaneously checked using the finite element analyses. The FE model of a tapered pipe was created using shell elements available in Autodesk Simulation Mechanical software. However, the use of FEA has some limitations, such as those regarding obtaining satisfactory results for a conical end of a pipe $\left(\vartheta =1\right)$ . The solid line in the graph shows a polynomial approximation of the ϑ−β relationship obtained analytically. A 6th degree polynomial provided an excellent agreement with the results, making it easy to find a correct value of the β -factor. The maximum difference of the values determined by the polynomial did not exceed $1.56\cdot 10^{-3}$ , which was completely sufficient in practical applications and experimental results. The equation of the polynomial used is (30):

(30) $$\beta \left(\vartheta \right)=\overset{6}{\underset{i=0}{\sum }}a_{i\;}\vartheta^{i},\;\;\;\;\;a=\left[\begin{array}{c} 1.8754728\\ 0.0551546\\ 0.8589286\\ -4.1604536\\ 9.9737731\\ -10.723535\\ 4.4254473 \end{array}\right]$$

The properties of the Sida hermaphrodita stem specimen can be assumed as: l=425 mm , $D_{1}=12.88\;\mathrm{mm}$ , $D_{2}=14.17\;\mathrm{mm}$ , g=1.77 mm , $\varrho =0.475\;\mathrm{mg}/{\mathrm{mm}}^{3}$ . Then, the tapering coefficient can be calculated: $\vartheta =1-D_{1}/D_{2}=0.09104,$ as well as the factor β=1.8851, using Equation (30). The cross-sectional area at the fixed end of the bar $A_{2}=\pi g\left(D_{2}-g\right)=68.805\;{\mathrm{mm}}^{2}$ and the moment of inertia ${ℑ}_{2}≅0.125\pi g{\left(D_{2}-g\right)}^{3}=1323.4\;mm^{4}$ together with the results of vibration frequency measurements (see Figure 1 and Table 4) allow us to estimate the Young’s modulus of the material. FFT analysis gives the first vibration frequency f=66.41 Hz and E=11.109 GPa, using Equation (2). The β -factor determined by solving Equation (29) directly is slightly larger, β=1.8854 , and produces a value of E=11.103 GPa . As shown, the polynomial approximation of the β -factor gives a result that is sufficiently exact when applied to the interpretation of the experimental data. Such an approach is convenient and reduces the results’ obtaining time. There is no need to use complex algorithms to solve Equation (29).

In Equation (3), the member responsible for the damping of vibrations is omitted. A negligible effect on the obtained vibration frequency can be proved by an example. An analysis of the signal enables us to find a logarithmic damping decrement δ , and hence a correction in Equation (2), resulting in the frequency of damped vibrations $\omega_{d}$ (31):

(31) $$\omega_{d}=2\pi f\sqrt{1-\alpha^{2}}\;\to \;E={\left(\frac{2\pi f}{\beta^{2}}\right)}^{2}\frac{\varrho Al^{4}}{ℑ\left(1-\alpha^{2}\right)}$$

where $\alpha =\delta /\sqrt{\delta^{2}-4\pi^{2}}$ is the damping ratio. In the described test sample, an estimated value of logarithmic damping decrement was equal to δ=0.1315 , which resulted in α=0.02093 , and the correction to the Young’s modulus value was $E^{\prime }=E/\left(1-\alpha^{2}\right)=1.0004E$ . As can be seen, the value of this correction is insignificant compared to the accuracy of the experimental results.

### 3.2. Tapered Pipe with Linearly Varying Thickness

The relationships presented in this section apply to such variations in the cross section, wherein the area changes according to the equation $A\left(x\right)=A_{0}x^{2}$ , and the moment of inertia to $ℑ\left(x\right)={ℑ}_{0}x^{4}$ . The variation parameter n=2 can be assumed not only for a truncated cone and tapered pipe with linearly varying thickness, but also for a pyramid. Details of these relationships can be found in Appendixes A.2 and A.3. Solving the problem allows us to find the eigenvalues of the matrix describing the boundary conditions (Equations (15) and (16)). This is analogous to that which was presented in the earlier section. The matrix describing the boundary conditions, for parameter n=2 , takes the form (32):

(32) $$B\left(\eta \right)=\left[\begin{array}{cccc} J_{2}\left(\eta \zeta_{2}\right) & Y_{2}\left(\eta \zeta_{2}\right) & I_{2}\left(\eta \zeta_{2}\right) & K_{2}\left(\eta \zeta_{2}\right)\\ J_{3}\left(\eta \zeta_{2}\right) & Y_{3}\left(\eta \zeta_{2}\right) & -I_{3}\left(\eta \zeta_{2}\right) & K_{3}\left(\eta \zeta_{2}\right)\\ J_{4}\left(\eta \zeta_{1}\right) & Y_{4}\left(\eta \zeta_{1}\right) & I_{4}\left(\eta \zeta_{1}\right) & K_{4}\left(\eta \zeta_{1}\right)\\ J_{5}\left(\eta \zeta_{1}\right) & Y_{5}\left(\eta \zeta_{1}\right) & -I_{5}\left(\eta \zeta_{1}\right) & K_{5}\left(\eta \zeta_{1}\right) \end{array}\right]$$

As before, by changing the section tapering parameter ϑ in the interval 0≤ϑ≤1 , the first eigenvalues $\eta_{1}$ of the B matrix and the β -factors using the condition (33) can be found:

(33) $$\det\left(B\left(\eta \right)\right)=0\;\to \;\eta_{1}\;\to \;\beta =\eta_{1}\sqrt{\vartheta }\;$$

As in the earlier paragraph, the classical equation $\cosh\left(\beta \right)\cos\left(\beta \right)+1=0$ was used for a constant section ( ϑ=0 ). A simplified equation was solved for the conical end of the bar when ϑ=1 : $J_{2}\left(2\eta \right)I_{3}\left(2\eta \right)+J_{3}\left(2\eta \right)I_{2}\left(2\eta \right)=0$ , whose solution is $\eta_{1}≅2.9528$ .

The results of the calculations are shown in Table 2, where, as before, the diameter $D_{1}$ and the thickness of the pipe at the “thinner” end $g_{1}$ are also given, assuming that $D_{2}=14\;\mathrm{mm}$ and $g_{2}=3\;\mathrm{mm}$ .

(34) $$\beta \left(\vartheta \right)=\overset{6}{\underset{i=0}{\sum }}b_{i\;}\vartheta^{i},\;\;\;\;\;b=\left[\begin{array}{c} 1.8749150\\ 0.4155574\\ -0.1076414\\ 1.6658948\\ -3.0310535\\ 2.7504146\\ -0.6147947 \end{array}\right]$$

**Figure 4:**
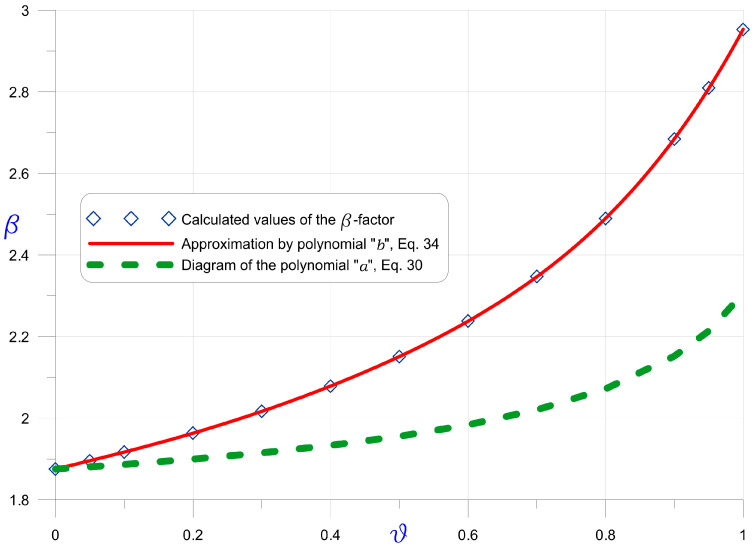
β -factor in dependence on the cone tapering coefficient ϑ for truncated cone (compare Figure A3), elliptical cone and solid prism (compare Figure A4) and pipe with linearly varying thickness (compare Figure A5).

Figure 4 shows the results of the β -factor calculations, in dependence on the tapering coefficient ϑ . The determined values were used to find an approximating polynomial “*b*” (Equation (34)). The approximating polynomial “*a*” described by Equation (30) is also shown to allow for comparison of the differences between the models of a pipe with constant thickness and a pipe with linearly decreasing thickness.

As before, an exemplary specimen’s data for Sida hermaphrodita was taken. The chosen bar corresponded best to a tapered pipe model with linearly decreasing thickness. Geometrical and material data were as follows: l=425 mm , $D_{1}=12.87\;\mathrm{mm}$ , $D_{2}=14.45\;\mathrm{mm}$ , $d_{1}=9.31\;\mathrm{mm}$ , $d_{2}=10.51\;\mathrm{mm}$ , $\varrho =0.489\;\mathrm{mg}/{\mathrm{mm}}^{3}$ . The cross-sectional area at the fixed end of the bar was $A_{2}=\pi g\left(D_{2}-g\right)=77.667{\;\mathrm{mm}}^{2}$ , and the moment of inertia was ${ℑ}_{2}≅0.125\pi g{\left(D_{2}-g\right)}^{3}=1547.1\;{\mathrm{mm}}^{4}$ . Next, the tapering coefficient could be calculated: $\vartheta =1-D_{1}/D_{2}=0.10934,$ and then the factor β=1.9209 according to Equation (34). FFT analysis gave the first vibration frequency, f=54.69 Hz , and finally E=6.9466 GPa using Equation (2). The β -factor determined by solving Equation (33) directly was slightly smaller, β=1.9208, and gave a value of E=6.9471 GPa . As before, it was convenient to use a polynomial approximation, which produces results very similar to the analytical solution.

### 3.3. The Use of Approximating Polynomials to Averaged Experimental Data

**Figure 5:**
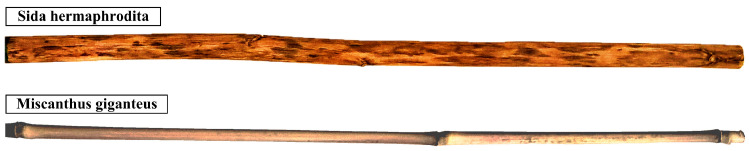
Naturally scaled Sida hermaphrodita and Miscanthus giganteus specimens with the same length.

As shown in Figure 5, when observing naturally scaled specimens, their geometry may vary significantly along the length. This can cause difficulties in obtaining an exact solution when using simplified cross-sectional properties approximations.

**Figure 6:**
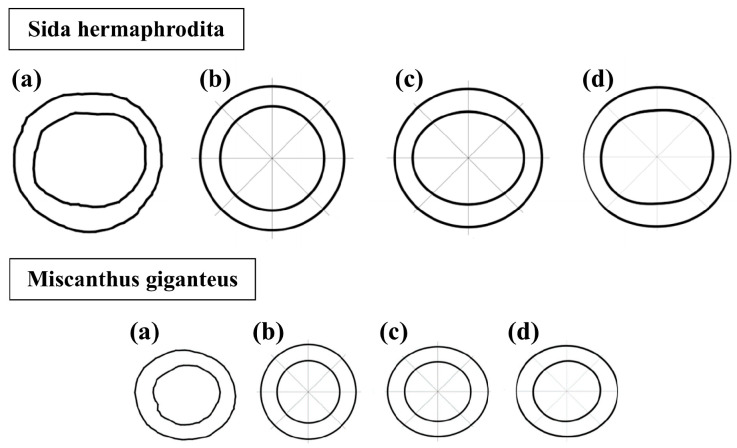
Possible cross-section approximations: (**a**) natural—no approximation, (**b**) circular pipe, (**c**) elliptical pipe, (**d**) sine–cosine series pipe.

As stated in an earlier work [2], several approximations of a cross section, presented in Figure 6, may be proposed, with different degrees of accuracy in calculations.

The most precise results were obtained when natural cross-sectional properties (Figure 6a) were used in calculations. This attempt necessitated generating a high-quality scan of the section slice, forming its vectorial contours and deciphering its mechanical properties directly from it. Nonetheless, the technique was impractical for utilisation in analytical approaches, due to many variables. The most accurate estimating technique was using sine–cosine series pipe (Figure 6d). This strategy introduced only a 2.3% error, compared to the natural section, with Sida hermaphrodita, and 1.7% error with Miscanthus giganteus. Similarly to the native section, using this approximation in analytical approaches can be very limited. Afterward, an elliptical pipe (Figure 6c) could be used with an averaged error at a level of 3.5% for Sida hermaphrodita and 3.3% for Miscanthus giganteus. This technique was significantly easier to implement in analytical approaches than the two techniques mentioned earlier. The most basic but less precise one was the circular pipe approximation (Figure 6b), which yielded a 3.6% error for Sida hermaphrodita and 7.5% for Miscanthus giganteus. However, this method can be effective in complex analytical approaches. In summary, the choice of approximation methods depends on the case being addressed. The one chosen in the paper was the circular pipe approximation, because of its ability to utilise a compound solution for vibration examinations of non-uniform cross-section cantilevers.

These inaccuracies can be substantially decreased when tests are done on a statistically valid number of specimens. The averaged properties of 10 samples tested for each plant in the experimental setup (Figure 1) were collected and are presented in Table 3. The percentage values in brackets are the averaged standard deviations of the measured characteristics.

An important issue can be an uncertainty estimation. This was done for frequencies found with FFT analysis. With a series of measurements, it is recommended to use the standard deviation of the experimental value of the mean. The variance of the mean can be used as a measure of uncertainty. An arithmetic mean is usually taken as the result of x measurement. Thus, the uncertainty of measurement is given by Formula (35):

(35) $$x=\bar{x}=\frac{\Sigma x_{i}}{n}\;\;\to \;\;s_{\bar{x}}=\sqrt{\frac{\Sigma {\left(x_{i}-\bar{x}\right)}^{2}}{n\left(n-1\right)}}$$

where:

x —measurement;
$\bar{x}$ —measurement mean;
n —number of measurements;
$s_{\bar{x}}$ —measurement uncertainty.

Table 4 presents FFT analyses results regarding the natural frequencies resulting from free-end oscillation measurements and properties for uncertainty estimation.

The measurement uncertainty of the FFT analyses of natural frequencies and relative error for Sida hermaphrodita stems can be estimated as (36):

(36) $$s_{\bar{x}}=\sqrt{\frac{\Sigma {\left(x_{i}-\bar{x}\right)}^{2}}{n\left(n-1\right)}}=\sqrt{\frac{173.95{\;\mathrm{Hz}}^{2}}{10\left(10-1\right)}}=1.39\;\mathrm{Hz},\frac{s_{\bar{x}}}{\bar{x}}=\frac{1.39\;\mathrm{Hz}}{64.48\;\mathrm{Hz}}=2.16\%$$

and for Micanthus giganteus stems as (37):

(37) $$s_{\bar{x}}=\sqrt{\frac{\Sigma {\left(x_{i}-\bar{x}\right)}^{2}}{n\left(n-1\right)}}=\sqrt{\frac{93.08{\;\mathrm{Hz}}^{2}}{10\left(10-1\right)}}=1.02\;\mathrm{Hz},\frac{s_{\bar{x}}}{\bar{x}}=\frac{1.02\;\mathrm{Hz}}{40.24\;\mathrm{Hz}}=2.53\%$$

As can be seen, the relative measurement error for both stems was slightly above 2%, which seems to be acceptable in a case such as that of a natural material.

The proposed β−ϑ relationships from Figure 3 and Figure 4 can be then directly utilised to calculate the modulus of elasticity of the vibrating cantilevers, based on Formula (2). Experimental levels of tapering were established using averaged values and their average standard deviations. To demonstrate the method for using the polynomials (30) and (34), three levels of tapering were compared:

- No tapering—using only one external diameter, as an average value from fixed support and free end;
- Average tapering—using two different outer diameters—one from the fixed support and another from the free end—averaged from the experimental data;
- High tapering—including average standard deviations of external diameters in such a way as to get the biggest diameter at the fixed support and the smallest at the free end.

The constant values used in these cases were f=64.84 Hz , l=426 mm , $\varrho =0.491\;\mathrm{mg}/{\mathrm{mm}}^{3}$ , $g=\left(g_{1}+g_{2}\right)/2=1.89\;\mathrm{mm}$ for Sida hermaphrodita, and f=40.23 Hz , l=392 mm , $\varrho =0.625\;\mathrm{mg}/{\mathrm{mm}}^{3}$ , $g=\left(g_{1}+g_{2}\right)/2=1.19\;\mathrm{mm}$ for Miscanthus giganteus. The results are provided in Table 5. The “1st approach” means that Figure 3 and Formula (30) were used, whereas the “2nd approach” indicates the use of Figure 4 and Formula (34).

As presented, the results may vary significantly depending on the assumed approach. To state which of them fits the best to the analysed natural plant stems, a verification by three-point bending test was used and is provided in the next subsection.

### 3.4. Validation of the Results by a Three-Point Bending Test

**Figure 7:**
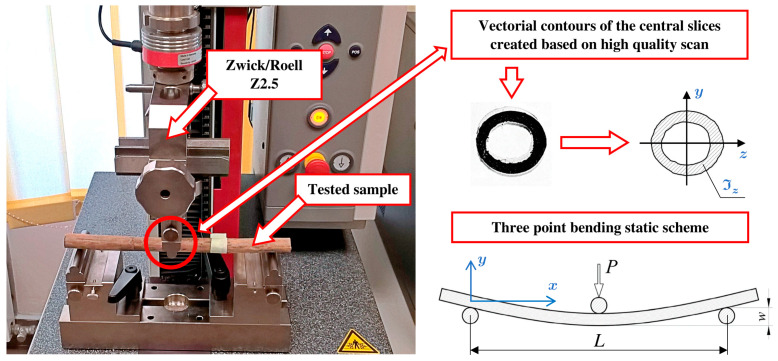
Three-point bending test done on Zwick/Roell Z2.5 testing machine.

The experiment which can verify the proposed method is a three-point bending test, standardised by ASTM D790 [31]. Such an evaluation, conducted on the Zwick/Roell Z2.5 testing machine, is depicted in Figure 7. The cross-sectional properties were obtained directly from the vectorial contours of the middle slices, and no shape approximations were executed, which should generate reliable results.

After a small rearrangement of a beam deflection formula, the modulus of elasticity could be calculated (38). P/w could be directly obtained as a slope of a force–deflection line from experiments.

(38) $$w=\frac{PL^{3}}{48E{ℑ}_{z}}\;\to \;E=\frac{L^{3}}{48{ℑ}_{z}}\frac{P}{w}$$

where:

w —deflection in the middle of the sample;
P —applied force;
L —span of the beam between supports;
E —modulus of elasticity;
${ℑ}_{z}$ —moment of inertia for central cross-section.

The complete examinations of 15 specimens were shown in earlier authors’ work [1]. Summarising the range of the modulus of elasticity for Sida hermaphrodita resulted in values of 7.63 ÷ 11.01; the average value was equal to 9.32 GPa with an 18% deviation. Subsequently, we found that the range of the modulus of elasticity for Miscanthus giganteus was 12.77 ÷ 15.93 GPa, with an average value equal to 14.35 GPa with an 11% deviation. As is visible, the spread of the results was considerable. 

Based on Table 6, one can see that results depend on the assumed method of calculations. Comparing results from the vibrations approaches to a three-point bending, the following statements can be made:

- The no-tapering assumption highly overestimated the value of the modulus of elasticity—39.2% error for Sida hermaphrodita and 16.0% for Miscanthus giganteus;
- Averaging tapering produced the best results for Miscanthus giganteus (4.7% error) and overestimated them for Sida hermaphrodita (16.6% error);
- High tapering gave perfect results for Sida hermaphrodita (0.0% error) and underestimated them for Miscanthus giganteus (11.1% error).

Based on the above, it can be generalised that the most exact approach for Sida hermaphrodita is the “high tapering” and for Miscanthus giganteus the “average tapering” assumption.

## 4. Conclusions

The article presented an original method for determining the modulus of elasticity of natural materials. A studied solution was based on the vibrations of non-uniform circular cross-section cantilevers and solved using Bessel functions. The derived equations, together with experimental tests, allowed for calculating the material’s properties. Assessments were based on measurements of the free-end oscillations in time using the Digital Image Correlation (DIC) method. They were induced manually and positioned at the end of the cantilever and monitored in time using a fast Vision Research Phantom v12.1 Camera with 1000 fps. GOM Correlate software tools were then used to find increments of deflection on a free end in every frame. It provided us with the ability to make diagrams containing a displacement–time relation. To find natural vibration frequencies, fast Fourier transform (FFT) analyses were conducted. The correctness of the proposed method was compared with a three-point bending test performed on a Zwick/Roell Z2.5 testing machine. Based on our experimental results comparison, it was stated that the most exact approach for Sida hermaphrodita was the “high tapering”, and for Miscanthus giganteus, the “average tapering” assumption. As was proved in the third section, the method generates trustworthy results and can confirm elastic properties of natural materials obtained in various experimental tests.

The factor of the specimens’ geometry deviating significantly from the ideal straight rod with a tapered cross section can cause a noticeable discrepancy when interpreting laboratory test results. Estimating the error interval obviously requires many trials and wider statistical analysis. The simple statistics presented in the paper were based on a mean and a standard deviation. They should be further extended, as shown by Giaccu et al. [32], who applied a sensitivity analysis to the elastic modulus of plywood determination. An imperfections effect caused by a natural curvature of the samples on the vibration frequencies was small, as verified by numerical models performed with the finite element method. The stiffening effect of nodes (diaphragms) visible on the Miscanthus giganteus stem specimen (Figure 5) requires a separate study, which the authors intend to perform. The shape of a cross section deviating significantly from the adopted circular one does not pose a problem when the specimens are treated as bent bars, because the stiffness depends on the cross-sectional moment of inertia. Such properties are easily determined based on cross-sectional slice scans after suitable graphic processing. Having both moments of inertia and principal axes, the section can be modelled with an ellipse, or elliptical tube, which belongs to the class of the section analysed in the work (see Appendix A.3). The non-uniform tapering of the outer and inner diameters of the pipe’s cross sections remains a considerable problem in modelling the stems. The Bessel functions cannot be used with differences in the two tapers. In such cases, only the numerical methods can be used, as Mabie and Rogers [12] did with doubly tapered rectangular cross-section bars. Finding suitable solutions and approximating polynomials for these cases is the goal of the authors’ further work.

## Figures and Tables

**Table 1:** Results of calculation of eigenvalues $\eta_{1}$ and β -factor for tapered pipe.

ϑ	$D_{1}$ (mm)	ε (%)	$x_{1}$	$\zeta_{1}$	$x_{2}$	$\zeta_{2}$	$\eta_{1}$	β
0	14.0	4.1	∞	∞	∞	∞	---	1.8751
0.05	13.3	4.5	19	8.7178	20	8.9443	8.4102	1.8806
0.10	12.6	5.0	9	6	10	6.3246	5.9655	1.8865
0.20	11.2	6.4	4	4	5	4.4721	4.2475	1.8996
0.30	9.8	8.3	2.3333	3.0551	3.3333	3.6515	3.4961	1.9149
0.40	8.4	11.3	1.5000	2.4495	2.500	3.1623	3.0566	1.9332
0.50	7.0	16.3	1	2	2	2.8284	2.7654	1.9555
0.60	5.6	25.5	0.6667	1.6330	1.6667	2.5820	2.5607	1.9835
0.70	4.2	45.4	0.4286	1.3093	1.4286	2.3905	2.4147	2.0203
0.80	2.8	---	0.2500	1	1.2500	2.2361	2.3164	2.0718
0.90	1.4	---	0.1111	0.6667	1.1111	2.1082	2.2683	2.1519
0.95	0.7	---	0.0526	0.4588	1.0526	2.0520	2.2711	2.2136
1	0	---	0	0	1	2	2.3055	2.3055

**Table 2:** Results of calculation of eigenvalues $\eta_{1}$ and β -factor for tapered pipe.

ϑ	$D_{1}$ (mm)	$g_{1}$ (mm)	$x_{1}$	$\zeta_{1}$	$x_{2}$	$\zeta_{2}$	$\eta_{1}$	β
0	14.0	3	∞	∞	∞	∞	---	1.8751
0.05	13.3	2.85	19	8.7178	20	8.9443	8.4760	1.8953
0.10	12.6	2.7	9	6	10	6.3246	6.0611	1.9167
0.20	11.2	2.4	4	4	5	4.4721	4.3903	1.9634
0.30	9.8	2.1	2.3333	3.0551	3.3333	3.6515	3.6820	2.0167
0.40	8.4	1.8	1.5000	2.4495	2.500	3.1623	3.2859	2.0782
0.50	7.0	1.5	1	2	2	2.8284	3.0414	2.1506
0.60	5.6	1.2	0.6667	1.6330	1.6667	2.5820	2.8894	2.2381
0.70	4.2	0.9	0.4286	1.3093	1.4286	2.3905	2.8054	2.3472
0.80	2.8	0.6	0.2500	1	1.2500	2.2361	2.7831	2.4893
0.90	1.4	0.3	0.1111	0.6667	1.1111	2.1082	2.8294	2.6842
0.95	0.7	0.15	0.0526	0.4588	1.0526	2.0520	2.8826	2.8096
1	0	0	0	0	1	2	2.9528	2.9528

**Table 3:** Averaged properties of Sida hermaphrodita and Miscanthus giganteus samples.

Material	$D_{2}$ (mm)	$d_{2}$ (mm)	$g_{2}$ (mm)	$D_{1}$ (mm)	$d_{1}$ (mm)	$g_{1}$ (mm)	l (mm)	ϱ (mg/mm^3^)	f (Hz)
Sida hermaphrodita	14.73 (4.8%)	10.88 (5.4%)	1.93 (6.1%)	13.23 (4.1%)	9.53 (4.3%)	1.85 (4.7%)	426 (1.1%)	0.491 (7.2%)	64.84 (4.3%)
Miscanthus giganteus	7.47 (4.6%)	5.01 (4.8%)	1.23 (6.9%)	7.04 (6.5%)	4.72 (5.4%)	1.16 (10.3%)	392 (2.3%)	0.625 (6.1%)	40.23 (6.4%)

**Table 4:** Result of FFT analyses of natural frequency and properties for uncertainty estimation.

Sample Number	Sida Hermaphrodita		Mischanthus Giganteus	
	f (Hz)	${\left(x_{i}-\bar{x}\right)}^{2}$ (Hz^2^)	f (Hz)	${\left(x_{i}-\bar{x}\right)}^{2}$ (Hz^2^)
1	62.50	5.49	39.06	1.37
2	54.69	103.15	37.11	9.77
3	70.31	29.91	42.97	7.48
4	64.45	0.15	37.11	9.77
5	66.41	2.44	39.06	1.37
6	66.41	2.44	41.02	0.61
7	64.45	0.15	42.97	7.48
8	64.45	0.15	46.88	44.10
9	70.31	29.91	37.11	9.77
10	64.45	0.15	39.06	1.37
	$\bar{x}=64.48$	Σ=173.95	$\bar{x}=40.24$	Σ=93.08

**Table 5:** Results for different approaches based on statistically valid experimental data.

				1st Approach	2nd Approach
Material	Level of Tapering	$D_{2}$ (mm)	$D_{1}$ (mm)	E (GPa)	E (GPa)
Sida hermaphrodita	No	13.98	13.98	12.961	12.977
	Average	14.73	13.23	11.223	10.515
	High	15.44	12.69	9.852	8.787
Miscanthus giganteus	No	7.25	7.25	16.630	16.650
	Average	7.47	7.04	15.310	14.739
	High	7.81	6.58	13.386	12.106

**Table 6:** Comparison of the averaged results from vibrations approaches and three-point bending.

Material	Level of Tapering	Vibrations E (GPa)	Three-Point Bending E (GPa)	Error (%)
Sida hermaphrodita	No	12.97	9.32	39.2
	Average	10.87		16.6
	High	9.32		0.0
Miscanthus giganteus	No	16.64	14.35	16.0
	Average	15.02		4.7
	High	12.75		11.1

**Figure A1:**
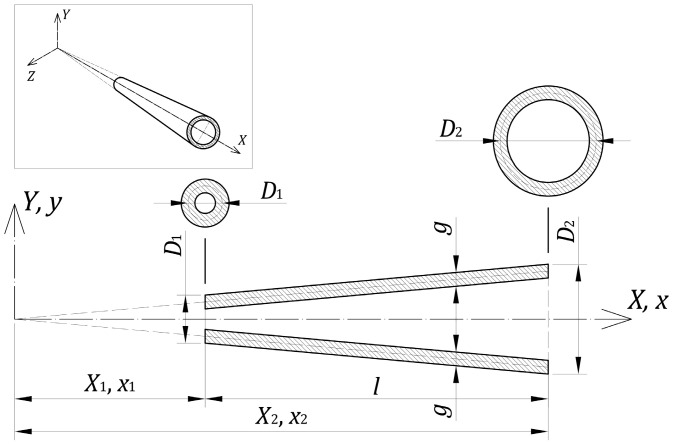
Constant-thickness pipe denotations.

**Figure A2:**
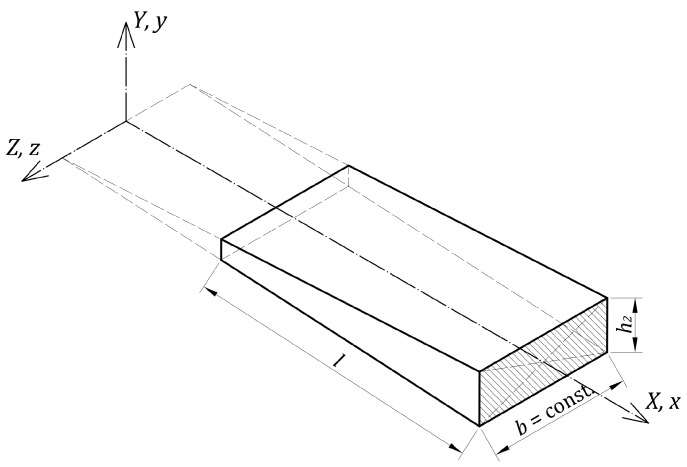
Constant width wedge.

**Figure A3:**
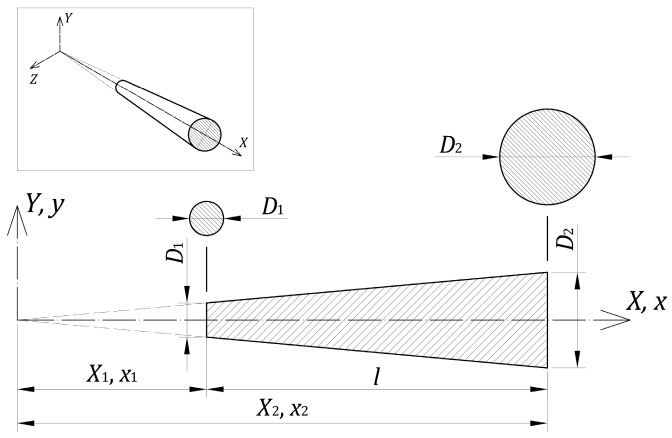
Solid truncated cone.

**Figure A4:**
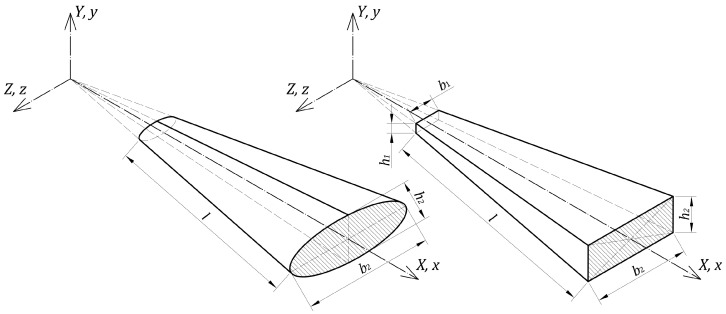
Elliptical cone and solid prism.

**Figure A5:**
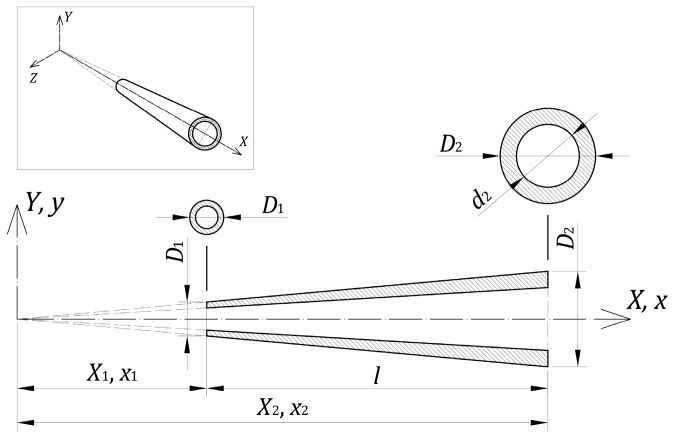
Pipe with linearly varying thickness.

## Data Availability

Not applicable.

## Appendix A

### Appendix A.1. Constant-Thickness Pipe

When $D_{1}$ and $D_{2}$ are the outer diameters of a pipe with a wall thickness of g , it can be assumed that (A1):

(A1)
$$A_{2}=\pi g\left(D_{2}-g\right)\;\to \;A_{0}=\frac{A_{2}}{x_{2}}\;\;\to \;A\left(x\right)=A_{0}x\;\;\to \;A\left(x\right)=A_{0}x^{n},\;\;n=1$$

For a pipe with a small and constant thickness, where $g\ll D\left(x\right)$ , a simplified formula to calculate the moment of inertia $ℑ\left(x\right)$ can be applied (A2):

(A2)
$${ℑ}_{2}≅\frac{\pi g}{8}{\left(D_{2}-g\right)}^{3}\;$$

Then (A3):

(A3)
$$\;{ℑ}_{0}=\frac{{ℑ}_{2}}{{\left(x_{2}\right)}^{3}}\;\;\to \;\;ℑ\left(x\right)=ℑx^{3}\;\to \;\;\;ℑ\left(x\right)={ℑ}_{0}x^{n+2},\;\;n=1$$

where:

D —cross-sectional diameter measured to the mid-thickness of the pipe;
A —cross-sectional area;
ℑ —moment of inertia;
g —thickness of the pipe wall.

The relative error of the estimation is proportional to $2{\left(g/D\right)}^{2}$ . For example, a pipe with a ratio D/g=5 produces a moment of inertia ${ℑ}_{2}$ , 8% smaller than the exact value.

For a wedge of constant width and linearly varying height, when $A_{2}$ is assumed as the cross-sectional area of the wedge at its thicker end and ${ℑ}_{2}$ as the moment of inertia of the cross section at this end (Figure A2), relations identical to Appendixes A1 and A3 are obtained.

(A4)
$$A_{2}=bh_{2}\;\;\to \;\;A_{0}=\frac{A_{2}}{x_{2}}\;\;\to \;\;A\left(x\right)=A_{0}x\;\;\to \;\;A\left(x\right)=A_{0}x^{n},\;\;n=1$$

(A5)
$$\;{ℑ}_{2}=\frac{bh_{2}{}^{3}}{12},\;{ℑ}_{0}=\frac{{ℑ}_{2}}{{\left(x_{2}\right)}^{3}}\;\;\to \;\;ℑ\left(x\right)={ℑ}_{0}x^{3}\;\to \;\;ℑ\left(x\right)={ℑ}_{0}x^{n+2},\;\;n=1$$

Equations (A4) and (A5) represent the variation of the cross-sectional area and moment of inertia of a wedge of constant width.

### Appendix A.2. Truncated Cone

(A6)
$$D\left(x\right)=D_{2}\left(\frac{x}{x_{2}}\right)\;$$

(A7)
$$A\left(x\right)=\frac{\pi }{4}{\left(\frac{D_{2}}{x_{2}}\right)}^{2}x^{2}\;\to \;A_{0}=\frac{\pi }{4}{\left(\frac{D_{2}}{x_{2}}\right)}^{2}\;\to \;\;A\left(x\right)=A_{0}x^{2}\;\;\to \;\;A\left(x\right)=A_{0}x^{n}$$

(A8)
$$ℑ\left(x\right)=\frac{\pi }{64}{\left(\frac{D_{2}}{x_{2}}\right)}^{4}x^{4}\;\to \;{ℑ}_{0}=\frac{\pi }{64}{\left(\frac{D_{2}}{x_{2}}\right)}^{4}\;\to \;\;ℑ\left(x\right)={ℑ}_{0}x^{n+2},\;\;n=2$$

where:

D —cross-sectional diameter measured at the mid-thickness of the pipe;
A —cross-sectional area;
ℑ —moment of inertia.

Similar relationships can also be obtained for a regular and truncated prism, when $A_{0}$ and ${ℑ}_{0}$ is assumed to be dependent on the dimensions of the prism base as follows (A9):

(A9)
$$A_{0}=\frac{bh}{{\left(x_{2}\right)}^{2}},\;\;{ℑ}_{0}=\frac{bh^{3}}{12{\left(x_{2}\right)}^{4}},$$

where b is the width and h is the height of the prism base. This type of cross-sectional variation can also include the elliptical cone (Figure A4) and tapered pipe with linearly varying thickness, which is described in the next section.

The corresponding formulas for the elliptical cone are given in Equation (A10):

(A10)
$$A_{2}=\frac{\pi b_{2}h_{2}}{4}\;\;\to \;A_{0}=\frac{A_{2}}{{\left(x_{2}\right)}^{2}},\;\;{ℑ}_{2}=\frac{\pi b_{2}h^{3}}{64}\;\;\to \;{ℑ}_{0}=\frac{{ℑ}_{2}}{{\left(x_{2}\right)}^{4}}.$$

### Appendix A.3. Tapered Pipe with Linearly Varying Thickness

(A11a)
$$A\left(x\right)=\frac{\pi \left(D_{2}^{2}-d_{2}^{2}\right)}{4x_{2}^{2}}x^{2}\;\;\to \;A_{0}=\frac{\pi \left(D_{2}^{2}-d_{2}^{2}\right)}{4x_{2}^{2}}\;\;\to \;\;A\left(x\right)=A_{0}x^{2}$$

(A11b)
$$A(x)\;=\;A_{0}x^{n},\;n=2$$

(A12a)
$$ℑ\left(x\right)=\frac{\pi \left(D_{2}^{4}-d_{2}^{4}\right)}{64x_{2}^{4}}x^{4}\;\;\to \;{ℑ}_{0}=\frac{\pi \left(D_{2}^{4}-d_{2}^{4}\right)}{64x_{2}^{4}}\;\;\to \;\;ℑ\left(x\right)={ℑ}_{0}x^{4}$$

(A12b)
$$ℑ(x)\;=\;{ℑ}_{0}x^{n+2},\;n=2$$

where:

$D_{2}$ —external diameter at the thick end;
$d_{2}$ —internal diameter at the thick end;
A —cross-sectional area;
ℑ —moment of inertia.
